# Cold Hands, Warm Heart: Quality of life, wellbeing, and mental health in Raynaud’s disease – An international survey study

**DOI:** 10.1177/13591053251407736

**Published:** 2026-01-16

**Authors:** Eleftheria Vaportzis

**Affiliations:** 1 University of Bradford, UK

**Keywords:** Raynaud’s disease, quality of life, wellbeing, mental health, international survey, temperature

## Abstract

The study assessed quality of life, wellbeing, and mental health in individuals living with Raynaud’s disease (RD), using a large international sample (*n* = 720). Participants completed Raynaud-Specific Quality of Life Questionnaire (RQLQ), WHO-5 Wellbeing Index, DASS-21 and demographics. Meteorological variables were recorded based on location. Participants with secondary RD reported lower quality of life and wellbeing, and higher anxiety, depression, and pain, than participants with primary RD or without a diagnosis. Participants without a diagnosis reported worse mental health than groups with a diagnosis. Participants in tropical climates reported the lowest quality of life, and those in temperate climates had the lowest wellbeing. Pain and symptom severity were the strongest predictors of quality of life. RD negatively affects quality of life, wellbeing, and mental health, particularly in secondary RD. Pain and symptom severity are key determinants. Meteorological factors contribute minimally. Tailored interventions focusing on symptom management should be prioritised.

## Introduction

Raynaud’s disease (RD) is a disorder of the peripheral vascular system characterised by episodes of pain, numbness, and colour changes in the extremities due to cold or stress. RD can be primary (pRD) or secondary (sRD) to an underlying condition such as scleroderma (Hughes and Herrick, 2016). The prevalence of pRD in the general population is around 5%, and although it varies by country (Garner et al., 2015), it carries a significant burden. It is estimated that pRD accounts for 80%–90% of RD cases, and sRD accounts for 10%–20% (National Institute for Health and Care Excellence, 2024). Although some studies found the prevalence of RD to be higher in colder climates (Plissonneau et al., 2015), other studies reported conflicting evidence (Pauling et al., 2019a; Purdie et al., 2009).

The impact of RD on quality of life depends on several factors, including the frequency, duration, and severity of episodes, as well as the presence of comorbidities (Merkel et al., 2002). sRD is progressive and can lead to irreversible damage (Curtiss et al., 2024). While pRD is considered benign, the pain, functional disability, and unpredictability of episodes (Hughes et al., 2015) lead individuals to make lifestyle changes (Merkel et al., 2002). Available treatments require daily routine modifications, are often ineffective, have side effects (Ture et al., 2024), and negatively impact individuals’ quality of life and mental health (Fábián et al., 2019).

Research investigating the impact of RD on quality of life is limited and has not always employed appropriate measures. Past research measured health-related quality of life using 36-Item Short Form Health Survey (SF-36; (Milio et al., 2006; Molnár et al., 2018)), the anxiety/depression dimension of EQ-5D (de Andrade et al., 2013), and measures of wellbeing (i.e. ONS4-Life Satisfaction; Broughton et al., 2024; Irving and Daniels, 2024). In the absence of RD-specific quality of life measures, Fábián et al. (2024) developed and validated the Raynaud Specific Quality of Life Questionnaire (RQLQ). Using RQLQ, Fábián et al. (2019) reported that people with sRD had significantly lower quality of life than those with primary RD.

People with RD report higher levels of stress (Irving and Daniels, 2024), anxiety (Broughton et al., 2024; Irving and Daniels, 2024), and depression (Broughton et al., 2024; Chen et al., 2024; Fábián et al., 2019; Irving and Daniels, 2024) compared with the general population. Some studies found those with sRD being more severely affected than those with pRD (Broughton et al., 2024; Fábián et al., 2019), while others found no differences between the two types (Irving and Daniels, 2024). Variations in findings may be explained by the different measures employed by these studies. A study with people with pRD reported that stress was not a significant predictor of RD episodes; however, they found a relationship between higher anxiety and a higher frequency of episodes at temperatures over 15.5°C (Brown et al., 2001). Surprisingly, this was the only study that investigated the role of temperature on psychological outcomes.

Previous studies reported mixed outcomes between cold temperatures and RD. Maricq et al. (1997) found that RD episodes were more likely to occur in colder climates. However, Pauling et al. (2019a) compared people with sRD in India and North America and reported that the difficulties they experienced in winter were not significantly different, despite the temperature difference between the two regions being more than 20°C. Additionally, a New Zealand study found that RD was more prevalent in the warmer region of the country (Purdie et al., 2009). The relationship between temperature and RD remains underexplored and warrants further investigation.

Given the international scope of this study, it is important to acknowledge that geographical and national differences can shape health-related quality of life and climate-related vulnerability. For example, differences in healthcare access, infrastructure, and climate adaptation policies across countries can exacerbate RD-related outcomes. In high-income countries, climate mitigation efforts may widen health inequalities, if not equitably designed (Hjelmskog et al., 2025). Moreover, national patterns in healthy life expectancy reflect underlying socioeconomic and environmental determinants, including climate exposure and chronic disease prevalence (The British Academy, 2024). These contextual factors highlight the need to consider both individual and regional variability.

The revised Wilson and Cleary model provides a theoretical model to understand better health-related quality of life in people living with chronic disease (Ferrans et al., 2005). According to this model, there are four main determinants of overall quality of life including biological function (e.g. abnormal vasoconstriction), symptoms (e.g. pain), functional status (e.g. ability to hold small objects), and general health perceptions (e.g. perception of RD severity). Characteristics of the individual (e.g. age, gender) and their environment (e.g. average temperatures) influence all determinants and quality of life.

The current study was guided by the revised Wilson and Cleary model and aimed to conduct a survey-based assessment of quality of life, wellbeing, and mental health in people living with RD. The study addressed gaps in the literature by employing an RD-specific measure for quality of life, using a large international sample, and including meteorological variables in the analyses.

## Methods

### Recruitment

A cross-sectional survey was designed, following guidelines by Kelley et al. (2003). Eligibility criteria included having a diagnosis or suspecting they have RD, being over 18 years old, and being fluent in English. The survey was distributed in hard copy and online via Online Surveys (www.onlinesurveys.ac.uk) between March and April 2025. This timeframe was selected to capture seasonal transition in both hemispheres, allowing for meaningful variation in meteorological conditions. In the Northern Hemisphere, this period marks the shift from winter to spring, with increasing temperatures and variable weather. In the Southern Hemisphere, it marks the shift from summer to autumn, with cooling temperatures and changing humidity. Weather data were recorded for the survey date and preceding 4 weeks to account for short-term climatic influences. Data collection continued until two consecutive days passed without any new completions. Duplicate entries were minimised using IP tracking and timestamp checks provided by the Online Surveys platform. Participants were recruited using multiple outreach methods targeting individuals with lived experience of RD. Recruitment materials were disseminated via social media posts (e.g. Facebook, X, Instagram) and community groups related to RD, chronic illness, and vascular health. The study was promoted through newsletters, websites, and mailing lists of RD-focused charities and patient organisations across 14 countries, including the UK, USA, Canada, Australia, New Zealand, Ireland, Malta, India, Malaysia, the Philippines, Singapore, Nigeria, Kenya, and South Africa. Examples of organisations contacted include Scleroderma & Raynaud’s UK, Raynaud’s Association, The Myositis Association, Lupus Australia, Chronic Pain Ireland, Arthritis NZ, Lupus Trust India, Arthritis Foundation Malaysia, Scleroderma Awareness Philippines, Autoimmune Diseases Singapore, Lupus Matters, and South Africa Rheumatism & Arthritis Association. These organisations were contacted directly and received study materials including a poster with the survey link for dissemination. Snowball sampling was encouraged, and participants were invited to share the survey with others who may be eligible. All recruitment materials included eligibility criteria, ethics approval reference, and contact details for the research team. Ethics approval was granted by the Humanities, Social, and Health Sciences Research Ethics Panel at the University of Bradford (E1301).

### Survey design

#### Demographics

Demographic questions include participants’ age, gender, race, employment, smoking habits, and location.

#### Meteorological variables

Based on participants’ location, the following meteorological variables were recorded: average temperature and temperature variation on the day the survey was completed and the preceding 4 weeks, along with precipitation, humidity, dew point, wind, and air pressure for the same 4-week period. The Universal Thermal Scale was used to classify participants’ locations from coldest to warmest based on the average temperatures in the preceding 4 weeks (Peel et al., 2007). Participants’ climate regions were classified using the Köppen–Geiger climate classification (Peel et al., 2007). The wind chill was calculated using the following formula (Osczevski and Bluestein, 2005):

$$\begin{array}{c} Wind\;Chill\;\left({}^{\circ}C\right)\;=\;13.12\;+\;0.6215T\;\\ -\;11.37{\left(V\;x\;1.609\right)}^{0.16}\\ +\;0.3965T{\left(V\;x\;1.609\right)}^{0.16} \end{array}$$

Where:

T = temperature in °C

V = wind speed in mph

#### Raynaud’s diagnosis

Participants were asked: ‘Have you been diagnosed with Raynaud’s?’ (Yes/No). If they answered ‘Yes’, they were asked: ‘If yes, is your Raynaud’s primary or secondary?’ (Primary/Secondary). If they answered ‘No’, they completed the Raynaud’s quiz, which is a five-item screening quiz developed by the Raynaud’s Association (2019; e.g. ‘Are your fingers or toes often cold, even indoors on a warm day and air conditioning?’, Yes/No). At least one ‘Yes’ suggests that the person may have RD. Total scores can range between 0 and 5.

#### Pain severity

Following previous studies (Brown et al., 2001; Fábián et al., 2024), pain severity was assessed using three questions from SF-36 (Ware and Sherbourne, 1992). These items were modified for RD. Participants were asked to report the following questions for the past 4 weeks: ‘How often did you experience pain as a result of your Raynaud’s?’ (1 = Never – 4 = Often); ‘How intense was the pain that you experienced as a result of your Raynaud’s?’ (1 = No pain – 4 = Severe pain); and ‘How much did pain as a result of your Raynaud’s interfere with your normal work (Including both outside the home and housework)?’ (1 = Not at all – 5 = Extremely). The final raw scores (ranging from 3 to 13) were rescaled to range from 5 to 100 according to the SF-36 manual. Higher scores indicate a higher degree of pain.

#### Symptom severity

Symptom severity was assessed with the question ‘How do you perceive the severity of your Raynaud’s?’. The question is rated on a 5-point Likert scale (1 = Not serious at all – 5 = Serious). Higher scores indicate higher symptom severity.

#### The Raynaud-Specific Quality of Life Questionnaire (RQLQ)

RQLQ (Fabian et al., 2024) is a validated measure that assesses the impact of RD on quality of life. Participants are presented with 29 statements rated on a 5-point Likert scale (1 = Strongly agree – 5 = Strongly disagree). Scores are calculated for 5 subscales: (1) Impaired Hand Function (e.g. ‘My illness limits my grip strength’); (2) Social Interaction (e.g. ‘I have to conceal the body parts affected by my illness’); (3) Emotional Burden (e.g. ‘I am concerned that my condition will worsen’); (4) Control (e.g. ‘I wear thicker clothes’); and (5) Sleep (e.g. During the night, I wake up due to numbness). Total scores can range between 29 and 145 with higher scores indicating better quality of life. Cronbach’s α for the overall scale in the current study was excellent (0.94).

#### WHO-5 Well-Being Index (WHO-5)

WHO-5 (Bech et al., 2003) is a validated measure of subjective mental wellbeing. Participants are asked five positively worded statements relating to the past 2 weeks (e.g. I have felt cheerful and in good spirits). Each statement is rated on a 6-point Likert scale (0 = At no time – 5 = All of the time). Total scores can range between 0 and 30 with higher scores indicating better mental wellbeing. Cronbach’s α in the current study was excellent (0.90).

#### The Depression, Anxiety, and Stress Scale – 21 items (DASS-21)

DASS-21 (Lovibond and Lovibond, 1995) is a validated measure that assesses feelings of depression, anxiety, and stress. It comprises three 7-item scales. Participants are asked to indicate whether each statement has applied to them during the last week. Responses are given using a 4-point Likert-type scale (0 = Does not apply to me at all − 3 = Applies to me a lot/most of the time). Scores are summed and multiplied by two. The total score for each scale ranges between 0 and 42. Higher scores indicate a higher level of the respective construct. The total DASS-21 score is the sum of the three scales and can range between 0 and 126 with higher scores indicating a higher level of general psychological distress. Cronbach’s α in the current study was excellent for the overall scale (0.94) and Depression (0.92), and good for Anxiety (0.80) and Stress (0.88).

### Data analysis

Statistical analyses were performed in SPSS v. 28 (IBM, 2021). Alpha was set at 0.05. Potential outliers were identified using pre-determined *Z*-score cut-offs (±3.29; (Tabachnick and Fidell, 2013). Statistically significant outliers were identified on the RQLQ domains Emotional Burden (*n* = 3) and Control (*n* = 7), and DASS-21 Anxiety scale (*n* = 4). Despite the high values, the scores were plausible and did not influence analyses.

Differences in continuous variables among the three groups were analysed using one-way ANOVA with Bonferroni correction, while Chi-square tests for independence were used to evaluate differences in categorical variables. Likelihood ratios were reported due to violations of Chi-square assumptions. Post-analysis was performed using adjusted standardised residuals.

Multivariate Analysis of Variance (MANOVA) was conducted to examine group differences in quality of life, wellbeing, stress, anxiety, and depression based on diagnosis type and climate classification. Post-hoc analyses were performed using one-way ANOVAs with Bonferroni correction. Welch’s MANOVA was reported where appropriate due to the violation of homogeneity of variance as indicated by Levene’s test. Post-hoc analyses were performed using Welch’s ANOVAs followed by Games-Howell tests. Bootstrapped MANOVAs (2000 samples, 95% CI) were calculated to account for the non-normal distribution of scores. The assumption of homogeneity of variance-covariance matrices was assessed using Box’s M test.

Bivariate correlations assessed the relationship between quality of life, wellbeing, stress, anxiety, depression, pain and symptom severity, and meteorological variables. Spearman’s correlations were used due to violations of the assumption of normality.

Hierarchical multiple regressions were conducted separately on quality of life and wellbeing to assess the variance accounted for by type of diagnosis, pain and symptom severity, mental health, and meteorological variables. Assumptions of linearity, normality, and homoscedasticity were assessed through visual inspection of scatterplots. Models were checked for outliers (standardised residuals ± 3, Cook’s distance > 1) and multi-collinearity (tolerance < 0.01, variance inflation factor > 10). Bootstrapped regression analyses were run due to violations of normality. The survey platform enforced a forced-response design, resulting in no missing data for the validated questionnaires. A small number of responses were excluded from climate-related analyses due to vague or missing location information, which prevented accurate temperature classification. These cases were retained in the dataset but excluded from specific analyses where relevant. No imputation or listwise deletion was applied.

A priori power analysis using G*Power (Faul et al., 2009) with α = 0.05 suggested that the sample had enough power (0.80) to detect a small effect (*f*^2^ = 0.02) as 628 participants were required.

## Results

A total of 721 participants completed the survey. One participant was removed (Raynaud’s quiz score was 0), leaving 720 participants in the analyses. The sample included 340 individuals with pRD, 274 individuals with sRD, and 106 individuals without a diagnosis. Group comparisons for demographic variables are presented in Table 1, with corresponding *p*-values indicating statistical significance across RD groups. The three groups significantly differed on age, *F*(2,712) = 9.32, *p* < 0.001, η^2^ = 0.05, country, χ^2^(16) = 47.11, *p* < 0.001, Cramér’s *V* < 0.001, and employment, χ^2^(4) = 30.48, *p* < 0.001, Cramér’s *V* < 0.001. There were no significant differences in gender, race, and smoking (*p* > 0.05; see Table 1).

**Table 1.** Descriptive information by group.

Characteristic	Primary RD *n* = 340 *n* (%)	Secondary RD *n* = 274 *n* (%)	Undiagnosed RD *n* = 106 *n* (%)	Total sample *N* = 720 *N* (%)	*p*
Age Range, M (SD)	18–80, 47.22 (12.97)	20–80, 51.1 (13.34)	18–77, 45.72 (13.01)	18–80, 48.49 (13.27)	<0.001
SF-36 M (SD)	65.24 (20.74)	66.82 (19.69)	57.61 (20.70)	64.72 (20.54)	<0.001
Gender					1.06
Female	321 (94.4)	258 (94.2)	92 (86.8)	671 (93.2)	
Male	18 (5.3)	15 (5.5)	12 (11.3)	45 (6.3)	
Other	1 (.3)	1 (.4)	2 (1.9)	4 (.6)	
Race					0.78
White	315 (92.6)	250 (91.2)	97 (91.5)	662 (91.9)	
Black	2 (.6)	2 (.7)	1 (.9)	5 (.7)	
Asian	8 (2.4)	10 (3.6)	5 (4.7)	23 (3.2)	
Mixed	8 (2.4)	5 (1.8)	3 (2.8)	16 (2.2)	
Other	4 (1.2)	6 (2.2)	0 (0.0)	10 (1.4)	
Country					<0.001
UK	167 (49.1)	112 (40.9)	66 (62.3)	345 (47.9)	
USA	131 (38.5)	81 (29.6)	22 (20.8)	234 (32.5)	
Canada	15 (4.4)	28 (10.2)	3 (2.8)	46 (6.4)	
Australia/New Zealand	8 (2.4)	22 (8.0)	3 (2.8)	33 (4.6)	
Other Europe	10 (2.9)	14 (5.1)	6 (5.7)	30 (4.2)	
Other America	3 (.9)	1 (.4)	3 (2.8)	4 (.6)	
Asia	3 (.9)	7 (2.6)	1 (.9)	13 (1.8)	
Africa	1 (.3)	3 (1.5)	2 (1.9)	9 (1.3)	
Smoking					0.18
Never smoked	235 (69.1)	165 (60.2)	61 (57.5)	461 (64.0)	
Used to smoke	82 (24.1)	91 (33.2)	36 (34.0)	209 (29.0)	
Smokes occasionally	5 (1.5)	5 (1.8)	4 (3.8)	14 (1.9)	
Smokes some days	4 (1.2)	1 (.4)	1 (.9)	6 (.8)	
Smokes daily	14 (4.1)	12 (4.4)	4 (3.8)	30 (4.2)	
Employment					<0.001
Economically active	267 (78.5)	162 (59.1)	82 (77.4)	511 (71.0)	
Economically inactive	72 (21.2)	111 (40.5)	24 (22.6)	207 (28.7)	
Climate					0.01
Tropical	4 (1.2)	8 (2.9)	4 (3.8)	8 (2.9)	
Dry	21 (6.2)	14 (5.1)	2 (1.9)	14 (5.1)	
Temperate	231 (67.9)	183 (66.8)	83 (78.3)	183 (66.8)	
Continental	78 (22.9)	63 (23.0)	15 (14.2)	63 (97.8)	
Symptom severity					<0.001
Serious	28 (7.8)	20 (7.2)	2 (1.7)*	50 (6.6)	
Quite serious	54 (15.1)	67 (24.0)**	12 (10.1)	133 (17.6)	
Moderately serious	120 (33.5)	111 (39.8)	31 (26.1)	262 (34.7)	
Mildly serious	102 (33.5)	69 (24.7)*	36 (30.3)	207 (27.4)	
Not serious at all	17 (5.0)	4 (1.5)	16 (15.1)	37 (5.1)	

* *p* < 0.05, **p < 0.01. *p*-values reflect results from one-way ANOVA for continuous variables (e.g. age) and Chi-square tests for categorical variables (e.g. gender). Missing responses may result in non-rounded values.

A Chi-square test revealed a significant association between type of diagnosis and symptom severity, χ^2^(8) = 44.11, *p* = 0.001, Cramér’s *V* < 0.001. Examination of adjusted standardised residuals indicated that participants with sRD were underrepresented in the ‘Not serious at all’ (*z* = − 3.70, *p* < 0.001) and ‘Mildly serious’ categories (*z* = − 2.10, *p* < 0.05), and overrepresented in the ‘Quite serious’ category (*z* = 2.9, *p* < 0.01). Participants without a diagnosis were overrepresented in the ‘Not serious at all’ category (*z* = 5.2, *p* < 0.001) and underrepresented in the ‘Serious category’ (*z* = −2.1, *p* < 0.05). These results indicate that participants with sRD tended to report more severe symptoms, while those without a diagnosis were more likely to report minimal severity. This pattern reinforces the clinical distinction between diagnostic groups and highlights the variability in perceived symptom burden.

Welch’s ANOVA showed significant group differences in pain severity, *F*(2,290) = 7.90, *p* < 0.001, η² = 0.04. Games-Howell post-hoc comparison tests indicated that participants with pRD (*p* = 0.01) and sRD (*p* < 0.001) experienced a higher degree of pain compared to participants without a diagnosis.

**Figure 1.**
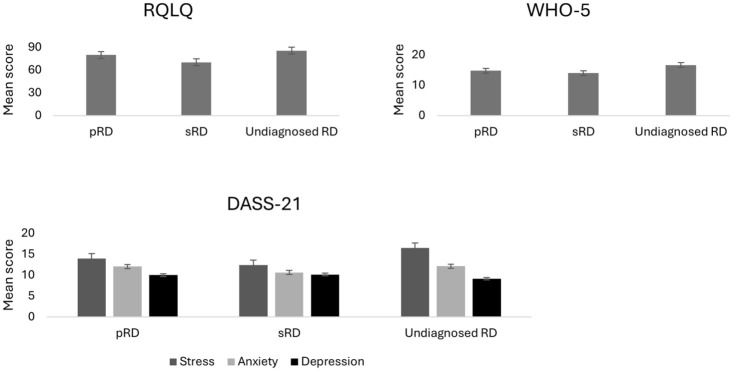
Mean scores on psychological outcomes by diagnostic group.

In comparison with normative data (Crawford and Henry, 2003), the sample had significantly higher scores on stress [pRD: *t*(339) = 8.22, *p* < 0.001, sRD: *t*(273) = 7.95, *p* < 0.001, undiagnosed: *t*(105) = 7.70, *p* < 0.001]; anxiety [pRD: *t*(339) = 13.3141, *p* < 0.001, sRD: *t*(273) = 12.63, *p* < 0.001, undiagnosed: *t*(105) = 10.42, *p* < 0.001], and depression [pRD: *t*(339) = 8.37, *p* < 0.001, sRD: *t*(273) = 7.33, *p* < 0.001, undiagnosed: *t*(105) = 8.53, *p* < 0.001]. A one-way MANOVA revealed a significant multivariate effect of type of diagnosis on quality of life, wellbeing, stress, anxiety, and depression, *F*(10, 1428) = 8.06, bootstrapped, *p* < 0.001, η^2^ = 0.05. Follow-up Welch’s ANOVA showed significant group differences on quality of life, *F*(2,717) = 27.15, bootstrapped *p* < 0.001, η^2^ = 0.11, and anxiety, *F*(2,717) = 4.18, bootstrapped *p* = 0.025, η^2^ = 0.05. Univariate ANOVAs showed significant group differences on wellbeing, *F*(2,717) = 11.91, bootstrapped *p* < 0.001, η^2^ = 0.03, stress, *F*(2,717) = 3.45, bootstrapped *p* = 0.03, η^2^ = 0.01, and depression, *F*(2,717) = 6.85, bootstrapped *p* < 0.001, η² = 0.03 (see Table 2 and Figure 1).

**Table 2.** Between-groups comparisons on dependent variables.

Variable	Primary RD *n* = 340 *n* (%)	Secondary RD *n* = 274 *n* (%)	Undiagnosed RD *n* = 106 *n* (%)	Total sample *N* = 720 *N* (%)	*p*
RQLQ Total	79.81 (20.46)	70.93 (17.42)	85.52 (20.74)	77.27 (20.09)	<0.001
WHO-5	16.85 (5.57)	14.76 (5.28)	16.65 (5.56)	16.02 (5.54)	<0.001
Stress M (SD)	13.66 (9.9)	13.92 (9.7)	16.45 (9.6)	14.17 (9.8)	0.03
Normal	204 (60.0)	167 (60.9)	53 (50.0)	424 (58.9)	
Mild	47 (13.8)	37 (13.5)	18 (17.0)	102 (14.2)	
Moderate	46 (13.5)	25 (9.1)	16 (15.1)	87 (12.1)	
Severe	24 (7.1)	34 (12.4)	8 (7.5)	66 (9.2)	
Extremely severe	19 (5.6)	11 (4.0)	11 (10.4)	41 (5.7)	
Anxiety M (SD)	9.45 (8.2)	10.60 (9.2)	12.13 (8.5)	10.28 (8.7)	0.02
Normal	157 (46.2)	122 (44.5)	28 (26.4)	307 (42.6)	
Mild	47 (13.8)	27 (9.9)	16 (15.1)	90 (12.5)	
Moderate	71 (20.9)	45 (16.4)	30 (28.3)	146 (20.3)	
Severe	21 (6.2)	30 (10.9)	12 (11.3)	63 (8.8)	
Extremely severe	44 (12.9)	50 (18.2)	20 (18.9)	114 (15.8)	
Depression M (SD)	9.94 (9.7)	10.18 (10.5)	13.94 (10.1)	10.62 (10.1)	0.001
Normal	194 (57.1)	156 (56.9)	40 (37.7)	390 (54.2)	
Mild	40 (11.8)	34 (12.4)	18 (17.0)	92 (12.8)	
Moderate	61 (17.9)	42 (15.3)	25 (23.6)	128 (17.8)	
Severe	21 (6.2)	16 (5.8))	9 (8.5)	46 (6.4)	
Extremely severe	24 (7.1)	26 (9.5)	14 (13.2)	64 (8.9)	

*p*-values reflect results from Welch’s ANOVA due to violations of homogeneity of variance. Post-hoc comparisons were conducted using Games-Howell tests. The cut-off points for each scale are: for Depression, 0–9 normal, 10–13 mild, 14–20 moderate, 21–27 severe, and 28+ extremely severe; for Anxiety, 0–7 normal, 8–9 mild, 10–14 moderate, 15–19 severe, and 20+ extremely severe; and for Stress, 0–14 normal, 15–18 mild, 19–25 moderate, 26–33 severe, and 34+ extremely severe.

Post-hoc tests showed that participants with sRD had significantly lower scores than those with pRD and without a diagnosis on quality of life (both *p* < 0.001), and wellbeing (*p* < 0.001, *p* = 0.01, respectively). Participants with primary RD had significantly lower scores than those without a diagnosis on quality of life (*p* = 0.04). Participants without a diagnosis had significantly higher stress (*p* = 0.3) and anxiety scores (*p* = 0.01) than those with pRD, and higher depression scores than those with pRD and sRD (both *p* = 0.01).

**Figure 2.**
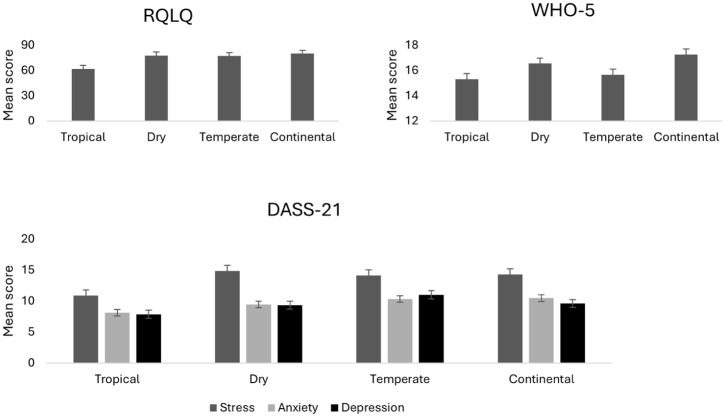
Mean scores for psychological outcomes across climate zones.

A one-way MANOVA revealed a significant multivariate effect of climate on quality of life and wellbeing, *F*(15, 2100) = 0.046, bootstrapped *p* = 0.01 partial η^2^ = 0.02. Follow-up univariate ANOVAs showed significant group differences on quality of life, *F*(3,702) = 4.08, bootstrapped *p* = 0.01, η^2^ = 0.017, and wellbeing, *F*(3,702) = 3.52, bootstrapped *p* = 0.02, η^2^ = 0.02. Post-hoc tests showed that participants living in tropical climates had significantly lower quality of life than those living in temperate (*p* = 0.02) and continental climates (*p* = 0.04). Additionally, participants in temperate climates had significantly lower wellbeing than those living in continental climates (*p* = 0.01; see Figure 2).

Bivariate correlations showed that quality of life, wellbeing, pain, and symptom severity were all significantly correlated in the expected directions (Table 3). Strong correlations were found between quality of life and pain (−0.551, *p* < 0.001), and symptom severity and pain (0.672, *p* < 0.001). There were also strong correlations between stress and anxiety (0.673, *p* < 0.001), stress and depression (0.715, *p* < 0.001), and anxiety and depression (0.648, *p* < 0.001; see Table 3).

**Table 3.** Spearman’s correlations between variables.

Variable	*1*	2	3	4	5	6	7	8	9	10	11	12	13	14
1. RQLQ Total score	–													
2. WHO-5	0.395**	–												
3. Stress	0.26	0.078*	–											
4. Anxiety	0.050	0.071	0.673**	–										
5. Depression	0.031	0.071	0.715**	0.648**	–									
6. SF-36	−0.551**	−0.303**	−0.049	−0.073	−0.026	–								
7. Symptom severity	−0.466**	−0.233**	−0.015	−0.018	−0.016	0.672**	–							
8. Fluctuation (4 weeks)	−0.001	0.074*	−0.041	−0.009	−0.54	−0.052	0.008	–						
9. Wind chill	−0.077*	−0.022	−0.010	0.035	0.083*	0.037	0.028	0.186**	–					
10. Precipitation	0.033	0.078*	0.070	0.051	0.083*	0.014	0.031	0.146**	0.292**	–				
11. Humidity	−0.034	−0.085*	0.025	0.010	0.053	0.052	−0.044	−0.757**	−0.135**	−0.172**	–			
12. Dew point	−0.112**	−0.066	0.005	0.041	0.114*	0.062	0.004	−0.161**	0.778*	0.226**	−0.287**	–		
13. Climate	0.083*	0.093*	0.024	0.035	−0.033	−0.075*	−0.027	0.150**	−0.516**	0.117**	−0.293**	−0.571**	–	
14. Temperature	−0.086*	0.012	0.018	0.022	0.061	0.062	0.027	0.250**	0.786**	0.332**	−0.187**	0.675**	−0.439**	–

Temperature classification according to the Universal Thermal Scale.

* *p* < 0.01. **p < 0.001.

In Step 1, the type of diagnosis explained 7% of the variance in quality of life, *R*^2^ = 0.07, *F*(1,659) = 50.78, *p* < 0.001. After entry in Step 2, pain and symptom severity explained an additional 30% of the variance, *R*^2^ change = 0.30, *F* change (2, 657) = 154.13, *p* < 0.001. In Step 3, wellbeing explained an additional 5% of the variance, *R*^2^ change = 0.05, *F change* (1, 656) = 50.28, *p* < 0.001. In Step 4, mental health variables did not account for a significant increase in the explained variance, *R*^2^ change = 0.001, *F* change (3, 656) = 0.20, *p* = 0.89. In Step 5, meteorological variables explained an additional 1% of the variance, *R*^2^ change = 0.01, *F* change (7, 646) = 2.16, *p* = 0.04. The total variance explained by the model was 42%, *R*^2^ = 0.42, *F*(7,646) = 2.16, *p* = 0.04 (see Table 4).

**Table 4.** Hierarchical regression analysis for quality of life.

Variable	Step 1			Step 2			Step 3			Step 4			Step 5		
	Unstandardised coefficients			Unstandardised coefficients			Unstandardised coefficients			Unstandardised coefficients			Unstandardised coefficients		
	*B* [95%, CI]	*SE B*	β	*B* [95%, CI]	*SE B*	β	*B* [95%, CI]	*SE B*	β	B [95%, CI]	SE B	β	B [95%, CI]	SE B	β
RD type	−7.90 [−10.08, 5.73]	1.11	−0.27**	−5.37 [−7.20, −3.54]	0.93	−0.18**	−4.57 [−6.35, −2.80]	0.91	−0.16**	−4.62 [−6.41, −2.83	0.91	−0.16**	−4.44 [−6.25, −2.64]	0.92	−15**
SF-36 Symptom severity				−0.41 [−0.48, −0.33] −3.90 [ −5.55, −2.25]	0.04 0.84	−0.41** −0.19**	−0.35 [−0.43, −0.27] −3.70 [−5.32, −2.14]	0.04 0.80	−0.39** −0.18**	−0.35 [−0.42, −0.27] −3.75 [−5.35, −2.16]	0.04 0.80	−0.35** −0.18**	−0.34 [−0.41, −0.26] −3.82 [−5.41, −2.23]	0.04 0.81	−0.34** 0.12**
WHO-5							0.82 [0.59, 1.04]	0.12	0.22**	0.82 [0.59, 1.04]	0.12	0.22**	0.83 [0.60, 1.06]	0.12	0.23**
Stress Anxiety Depression										0.05 [−0.14, 0.25] 0.02 [−0.19, 0.23] −0.07 [−0.25, 0.12]	0.10 0.11 0.09	0.02 0.01 −0.03	0.04 [−0.16, 0.24] 0.03 [−0.18, 0.24] −0.06 [−0.24, 0.13]	0.10 0.11 0.09	0.02 0.01 −0.03
Fluctuation (4 weeks) Wind chill Precipitation Humidity Dew point climate Temperature													−0.83 [−1.53, −0.12] 1.01 [0.07, 1.94] −0.02 [−0.06, 0.023] 0.11 [−0.20, 0.42] −1.1 [−2.28, 0.02] 0.97 [−1.67, 3.61] −2.27 [−6.75, 2.21]	0.36 0.37 0.02 0.16 0.58 1.34 2.28	−0.10* 0.37* −0.03 0.05 −0.31 0.03 −0.09
*R*^2^ change *F*^2^-change	0.07 50.78**			0.30 154.13**			0.05 50.28**			0.001 0.20			0.01 2.16*		

95% bootstrapped confidence interval [CI] based on 2000 samples. Temperature classification according to the Universal Thermal Scale.

* *p* < 0.05. **p < 0.001.

In Step 1, the type of diagnosis explained 3% of the variance in wellbeing, *R*^2^ = 0.03, *F*(1,659) = 17.39, *p* < 0.001. After entry in Step 2, pain and symptom severity explained an additional 8% of the variance, *R*^2^ change = 0.08, *F* change (2, 657) = 29.01, *p* < 0.001. In Step 3, quality of life explained an additional 6% of the variance, *R*^2^ change = 0.06, *F change* (1, 656) = 50.28, *p* < 0.001. In Step 4, mental health variables did not account for a significant increase in the explained variance, *R*^2^ change = 0.004, *F* change (3, 656) = 0.10, *p* = 0.39. In Step 5, meteorological variables explained an additional 2% of the variance, *R*^2^ change = 0.02, *F* change (7, 646) = 2.15, *p* = 0.04. The total variance explained by the model was 17%, *R*^2^ = 0.17, *F*(7,646) = 2.16, *p* = 0.04 (see Table 5).

**Table 5.** Hierarchical regression analysis for wellbeing.

Variable	Step 1			Step 2			Step 3			Step 4			Step 5		
	Unstandardised coefficients			Unstandardised coefficients			Unstandardised coefficients			Unstandardised coefficients			Unstandardised coefficients		
	*B* [95%, CI]	*SE B*	β	*B* [95%, CI]	*SE B*	β	*B* [95%, CI]	*SE B*	β	*B* [95%, CI]	*SE B*	β	*B* [95%, CI]	*SE B*	β
RD type	−1.30 [−1.90, −0.69]	0.31	−0.16**	−0.98 [−1.58, −0.39]	0.30	−0.12**	−0.51 [−1.10, 0.08]	0.30	−0.06	−0.47 [−1.06, 0.11]	0.30	−0.06	−0.58 [1.18, 0.02]	0.30	−07
SF-36 Symptom severity				−0.07 [−0.09, −0.04] −0.21 [−0.75, 0.32]	0.01 0.27	−0.26** −0.04	−0.035 [−0.60, −0.01] 0.15 [−0.40, 0.65]	0.01 0.26	−0.13* 0.03	−.0.3 [−0.06, −0.01] 0.12 [−0.41, 0.64]	0.01 0.27	−0.13* 0.02	−0.34 [−0.41, −0.26] −3.82 [−5.41, −2.23]	0.04 0.81	−0.34* 0.12
RQLQ total							0.09 [0.06, 0.11]	0.01	0.32**	0.09 [0.06, 0.11]	0.01	0.32**	0.83 [0.60, 1.06]	0.12	0.23**
Stress Anxiety Depression										0.02 [−0.05, 0.08] −0.01 [−0.08, 0.06] 0.02 [−0.04, 0.08]	0.03 0.04 0.03	0.03 −0.01 0.04	0.02 [−0.05, 0.08] −0.01 [−0.08, 0.06] 0.02 [−0.04, 0.08]	0.03 0.04 0.03	0.03 −0.01 0.04
Fluctuation (4 weeks) Wind chill Precipitation Humidity Dew point Climate Temperature													0.11 [−1.20, 0.34] −0.17 [−0.47, 0.13] 0.01 [0.00, 0.023]* −0.06 [−0.16, 0.05] 0.15 [−0.23, 0.52] 0.46 [−40, 1.32]] 0.60 [−0.86, 2.06]	0.12 0.15 0.01 0.05 0.19 0.44 0.74	0.05 −0.23 0.08 −0.09 0.15 0.05 0.09
*R*^2^ change *F*^2^-change	0.07 17.39**			0.08 29.01**			0.06 50.28**			0.004 0.10			0.02 2.15*		

95% bootstrapped confidence interval [CI] based on 2000 samples. Temperature classification according to the Universal Thermal Scale.

* *p* < 0.05, **p < 0.001.

## Discussion

This study assessed quality of life, wellbeing, and mental health in RD using a large, international sample. It addressed an important gap in the literature by adding various meteorological variables in the analyses and using an RD-specific measure for quality of life. It is the first study to include people without an RD diagnosis. Despite the relatively high prevalence of RD, it is frequently underdiagnosed due to a lack of diagnostic tools, lack of awareness, or symptom misinterpretation (SRUK, 2025; Temprano, 2016).

The findings are consistent with a body of research showing that mental health is worse in people with RD compared with the general population (Crawford and Henry, 2003). In line with past research participants with sRD reported lower quality of life and wellbeing (Fábián et al., 2019; Irving and Daniels, 2024), higher anxiety and depression (Broughton et al., 2024; Fábián et al., 2019), and greater pain and symptom severity than those with pRD or without a diagnosis (Broughton et al., 2024; Fábián et al., 2019). A cross-sectional survey study found that people with systemic and rheumatologic diseases were amongst those reporting the poorest quality of life (Bogart and Irvin, 2017). Interestingly, participants without a diagnosis reported lower mental health than the other groups. One possible explanation is that the lack of diagnosis contributes to poor mental health. Mund et al. (2023) found high rates of mental disorders in individuals seeking diagnosis for unexplained symptoms possibly due to uncertainty and associated frustration. In addition, a scoping review (Massazza et al., 2023) found that uncertainty is consistently associated with increased anxiety, stress, and depressive symptoms across diverse populations. Another explanation is that without a formal diagnosis individuals may have limited access to treatment or support. Clinicians should prioritise early screening, diagnosis, and tailored support for people living with systemic and rheumatologic diseases.

Participants living in tropical climates reported the lowest quality of life, while those living in temperate climates reported the lowest wellbeing. These findings challenge the notion that people living in colder climates experience RD more severely. People living in warmer climates may experience more frequent and rapid temperature changes due to air conditioning. Virgili-Gervais et al. (2024) reported that extreme heat can exacerbate RD symptoms due to rapid vasoconstrictive responses. Similarly, those living in temperate climates often experience extreme weather changes or conditions, making planning and prevention difficult (Legg, 2021). The impact of climate on RD may be more complex than a cold/hot dichotomy. Individual (e.g. adaptive and coping strategies) and societal factors (e.g. healthcare access, infrastructure) should be considered. While the findings suggest a potential association between climate and RD-related quality of life, these results should be interpreted with caution as the sample sizes for tropical climates were relatively small.

Although meteorological data were recorded objectively, individuals’ subjective experiences of temperature may differ from environmental conditions. Perceived cold sensitivity, thermal discomfort, and personal coping responses may influence symptom severity and quality of life beyond what is captured by objective meteorological data alone (Pauling et al., 2019b). In addition, it is possible that temperature variability, rather than absolute temperature, plays a more influential role in shaping RD experiences. Individuals living in tropical or temperate climates may still encounter abrupt or extreme temperature changes due to factors such as air conditioning, or seasonal transitions. These fluctuations may exacerbate symptoms and impact wellbeing. Subjective assessments of temperature variability offer valuable insight into the lived experience of RD, complementing objective meteorological data and helping to contextualise symptom severity and wellbeing outcomes.

Pain and symptom severity accounted for 30% of the variance in quality of life supporting the revised Wilson Cleary model, according to which symptoms and general health perceptions are determinants of overall quality of life. The strong correlation between pain and symptom severity highlights their role in influencing patients’ experiences. Mental health variables did not significantly account for an additional variance in either quality of life or wellbeing. Measures of quality of life or wellbeing may capture mental health indirectly (Topp et al., 2015) or there is an overlap between physical symptoms and mental health symptoms (Hanna et al., 2024). Meteorological variables accounted for up to 2% of the variance in quality of life and wellbeing, suggesting that although they are important, physical variables are more important. Interventions targeting pain and symptom management should be prioritised to improve the quality of life and wellbeing of people living with RD. Pain and symptom relief may also benefit mental health. While meteorological variables matter, they should complement rather than replace other assessments.

### Strengths, limitations and future research

This study employed a large international sample and validated instruments, including an RD-specific measure for quality of life. The inclusion of undiagnosed individuals and meteorological variables enhances ecological validity and captures the real-world diversity in RD experiences.

The study’s cross-sectional design does not allow us to infer how the relationships between the variables may differ over time. Self-reported data introduce biases including subjective interpretation of symptoms. Although including participants without a diagnosis is important given the underdiagnosis of RD, it is possible that some of those included did not have RD.

Recruitment via social media and RD charities likely attracted individuals who are more symptomatic or engaged, introducing self-selection bias. The sample’s predominance of white females further limits generalisability, especially given potential differences in sRD presentation across gender and race. Medication use was not assessed, which may have influenced symptom severity and psychological outcomes.

The mental health outcomes reported by participants without a diagnosis may have been influenced by other comorbid physical or mental health conditions (e.g. chronic illness, anxiety, depression). As the survey did not include screening questions for comorbidities, and sampling was conducted online, this remains a plausible factor that may have affected responses and should be considered when interpreting the findings. The Raynaud’s quiz was administered only to participants without a formal diagnosis. While this approach helped identify potential undiagnosed cases, it did not allow for verification of self-reported diagnoses. Screening tools should be administered to all participants to enhance diagnostic reliability, particularly in online survey designs.

Although objective meteorological data were used to classify climate exposure, the study did not assess participants’ subjective experiences of temperature or actual cold exposure. For instance, individuals living in colder climates may spend most of their time in warm, controlled indoor environments, while others in warmer regions may experience abrupt temperature drops due to air conditioning. These variations in real-world exposure and perception may influence symptom severity and wellbeing but were not captured in the current design.

Pharmacological data should be included to isolate the effects of RD from improvements related to medication. Several other variables may have affected the outcomes and should be considered (e.g. healthcare access, coping strategies). Longitudinal designs are needed to establish the directionality of influences and include clinical samples.

The RQLQ was originally developed and validated in Hungarian, and no English-language validation study currently exists. Although the measure demonstrated excellent internal consistency in the present study, the absence of formal English validation may limit generalisability and warrants further psychometric evaluation.

In this study, reliability was supported through forced-response design, IP tracking, and timestamp checks to minimise missing data and duplicate entries. Visual aids could enhance diagnostic accuracy and symptom reporting in online studies. For example, image-based prompts showing typical RD colour changes or hand postures may help participants better identify their symptoms. Dinsdale et al. (2025) developed a smartphone app that allowed patients to document RD episodes using photographic images, demonstrating the feasibility of visual data collection in RD research. Such approaches could be adapted for survey-based studies to enhance self-report reliability.

## Conclusion

This study showed that diagnosis type, pain, and symptom severity significantly affect quality of life, wellbeing, and mental health, while meteorological variables play a smaller role. Individuals with sRD experience the greatest burden including greater pain, and poorer quality of life and mental health. Tailored interventions targeting pain and symptom severity, are critical for improving patients’ quality of life and wellbeing. Findings related to climate should be interpreted with caution due to small subgroup sizes. These results are exploratory and warrant replication with more balanced samples.
